# Comparison of Glidescope Core, C-MAC Miller and conventional Miller laryngoscope for difficult airway management by anesthetists with limited and extensive experience in a simulated Pierre Robin sequence: A randomized crossover manikin study

**DOI:** 10.1371/journal.pone.0250369

**Published:** 2021-04-22

**Authors:** Andreas Moritz, Luise Holzhauser, Tobias Fuchte, Sven Kremer, Joachim Schmidt, Andrea Irouschek

**Affiliations:** Department of Anesthesiology, Faculty of Medicine, University Hospital Erlangen, Friedrich-Alexander-Universität Erlangen-Nürnberg, Erlangen, Germany; Kaohsuing Medical University Hospital, TAIWAN

## Abstract

**Background:**

Video laryngoscopy is an effective tool in the management of difficult pediatric airway. However, evidence to guide the choice of the most appropriate video laryngoscope (VL) for airway management in pediatric patients with Pierre Robin syndrome (PRS) is insufficient. Therefore, the aim of this study was to compare the efficacy of the Glidescope^®^ Core^™^ with a hyperangulated blade, the C-MAC^®^ with a nonangulated Miller blade (C-MAC^®^ Miller) and a conventional Miller laryngoscope when used by anesthetists with limited and extensive experience in simulated Pierre Robin sequence.

**Methods:**

Forty-three anesthetists with limited experience and forty-three anesthetists with extensive experience participated in our randomized crossover manikin trial. Each performed endotracheal intubation with the Glidescope^®^ Core^™^ with a hyperangulated blade, the C-MAC^®^ with a Miller blade and the conventional Miller laryngoscope. “Time to intubate” was the primary endpoint. Secondary endpoints were “time to vocal cords”, “time to ventilate”, overall success rate, number of intubation attempts and optimization maneuvers, Cormack-Lehane score, severity of dental trauma and subjective impressions.

**Results:**

Both hyperangulated and nonangulated VLs provided superior intubation conditions. The Glidescope^®^ Core^™^ enabled the best glottic view, caused the least dental trauma and significantly decreased the “time to vocal cords”. However, the failure rate of intubation was 14% with the Glidescope^®^ Core^™^, 4.7% with the Miller laryngoscope and only 2.3% with the C-MAC^®^ Miller when used by anesthetists with extensive previous experience. In addition, the “time to intubate”, the “time to ventilate” and the number of optimization maneuvers were significantly increased using the Glidescope^®^ Core^™^. In the hands of anesthetists with limited previous experience, the failure rate was 11.6% with the Glidescope^®^ Core^™^ and 7% with the Miller laryngoscope. Using the C-MAC^®^ Miller, the overall success rate increased to 100%. No differences in the “time to intubate” or “time to ventilate” were observed.

**Conclusions:**

The nonangulated C-MAC^®^ Miller facilitated correct placement of the endotracheal tube and showed the highest overall success rate. Our results therefore suggest that the C-MAC^®^ Miller could be beneficial and may contribute to increased safety in the airway management of infants with PRS when used by anesthetists with limited and extensive experience.

## Introduction

Pierre Robin syndrome (PRS), which occurs in approximately 1:5000–1:85000 live births [1], is characterized by the clinical triad of micrognathia, glossoptosis and upper airway obstruction, and is often associated with cleft palate [2]. Pediatric patients with PRS require general anesthesia for a variety of surgical procedures including tongue lip adhesion, mandibular distraction osteogenesis, tracheostomy and gastrostomy tube placement [1]. Due to the congenital craniofacial anomalies, airway management for general anesthesia or acute respiratory distress in uncorrected PRS patients can present significant challenges even for experienced anesthesiologists. Mao and colleagues reported difficult intubation in 57% of patient with PRS requiring mandibular distraction osteogenesis [3]. In a retrospective chart review including 51 infants with mandibular hypoplasia, Frawley and colleagues described an incidence of difficult intubation of even 71% [4]. Airway management problems are a major contributor to anesthesia associated morbidity and mortality in pediatric patients [5, 6]. In addition, multiple intubation attempts in children with difficult tracheal intubation have been shown to correlate with a higher rate of failure and an increased incidence of severe complications including hypoxemia and cardiac arrest [7]. Therefore, for difficult pediatric airway management, it is imperative to use the most appropriate airway device to increase the probability of success on the first tracheal intubation attempt.

Although fiberoptic-guided intubation under spontaneous breathing is considered the gold standard for anticipated difficult airway management [8], its use in small infants or in potentially uncooperative older children may be challenging. In recent years, however, several video laryngoscopes (VLs) suitable for pediatric patients have been developed and implemented in clinical practice. Due to different blade sizes, these devices can also be used for the management of a difficult airway in infants and newborns [8, 9]. In addition, VLs, including the C-MAC^**®**^ [10], the Airtraq^®^ [11], the McGrath MAC^™^ [12] and the Glidescope^®^ [13] have been successfully used in pediatric patients with PRS. In a randomized multi-institutional crossover study, Fiadjoe and colleagues even reported comparable first-attempt intubation success of Glidescope^®^ Cobalt video laryngoscopy and fiberoptic bronchoscopy when used in a Pierre Robin manikin [14].

Video laryngoscopy has been shown to be an effective tool in the management of difficult pediatric airway. However, evidence to guide the choice of the most appropriate VL for difficult intubation in infants with Pierre Robin sequence is insufficient. In addition, no study has yet compared the performance of hyperangulated and nonangulated VLs when used by experienced and inexperienced intubators in PRS patients. Thus, the aim of our prospective randomized crossover study was to compare the efficacy of the Glidescope^®^ Core^™^ VL with a hyperangulated blade (Glidescope^®^ Core^™)^, the C-MAC^®^ VL with a Miller blade (C-MAC^®^ Miller) and the conventional Miller laryngoscope when used by anesthetists with limited and extensive previous experience in a simulated Pierre Robin sequence.

## Materials and methods

### Study design and setting

The present randomized crossover manikin study was evaluated and approved by the institutional ethics committee (Ethics Committee of the Friedrich-Alexander-Universität Erlangen-Nürnberg; reference number: 408_18 B). Following written informed consent, eighty-six anesthetists were recruited to the study. Data were anonymized and information on the performance of individual participants was not made available to anybody outside the research team.

**Fig 1:**
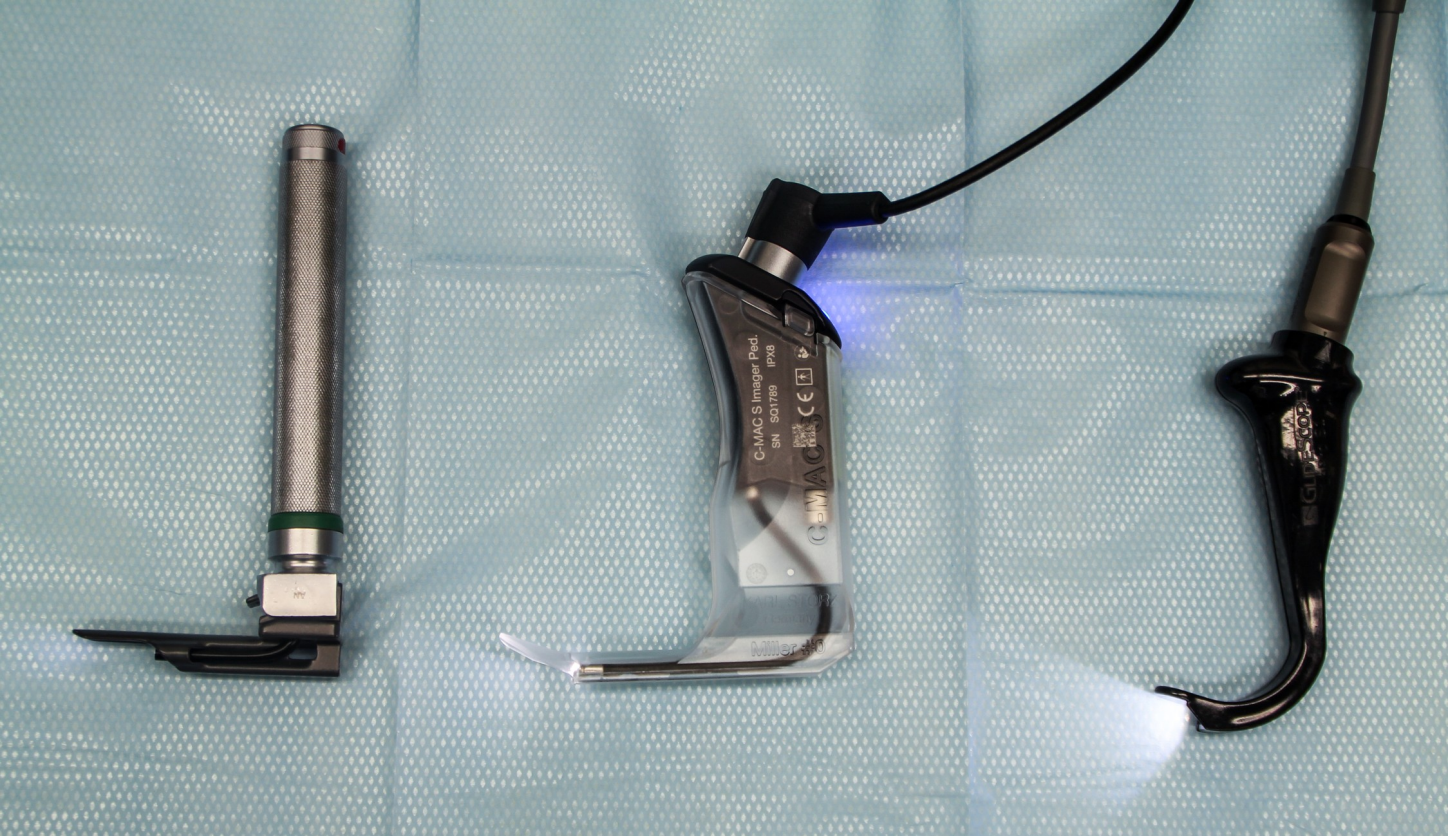
The three different airway devices used in this study. From left to right: Conventional Miller laryngoscope blade size 0 (Rüsch^®^ Polaris^™^ Single-Use Laryngoscope Blade Miller 0, Teleflex Medical Europe Ltd, Athlone, Ireland; Heine F.O. SLIM LED metallic laryngoscope handle, Heine Optotechnik GmbH & Co. KG, Gilching, Germany); C-MAC^®^ 8403 ZX VL with a single-use C-MAC S video laryngoscope blade Miller size 0 (Karl Storz, Tuttlingen, Germany); Glidescope^®^ Core^™^ 10 VL with a hyperangulated LoPro S1 single-use blade (Verathon Medical Canada ULC, Burnaby, BC, Canada).

**Fig 2:**
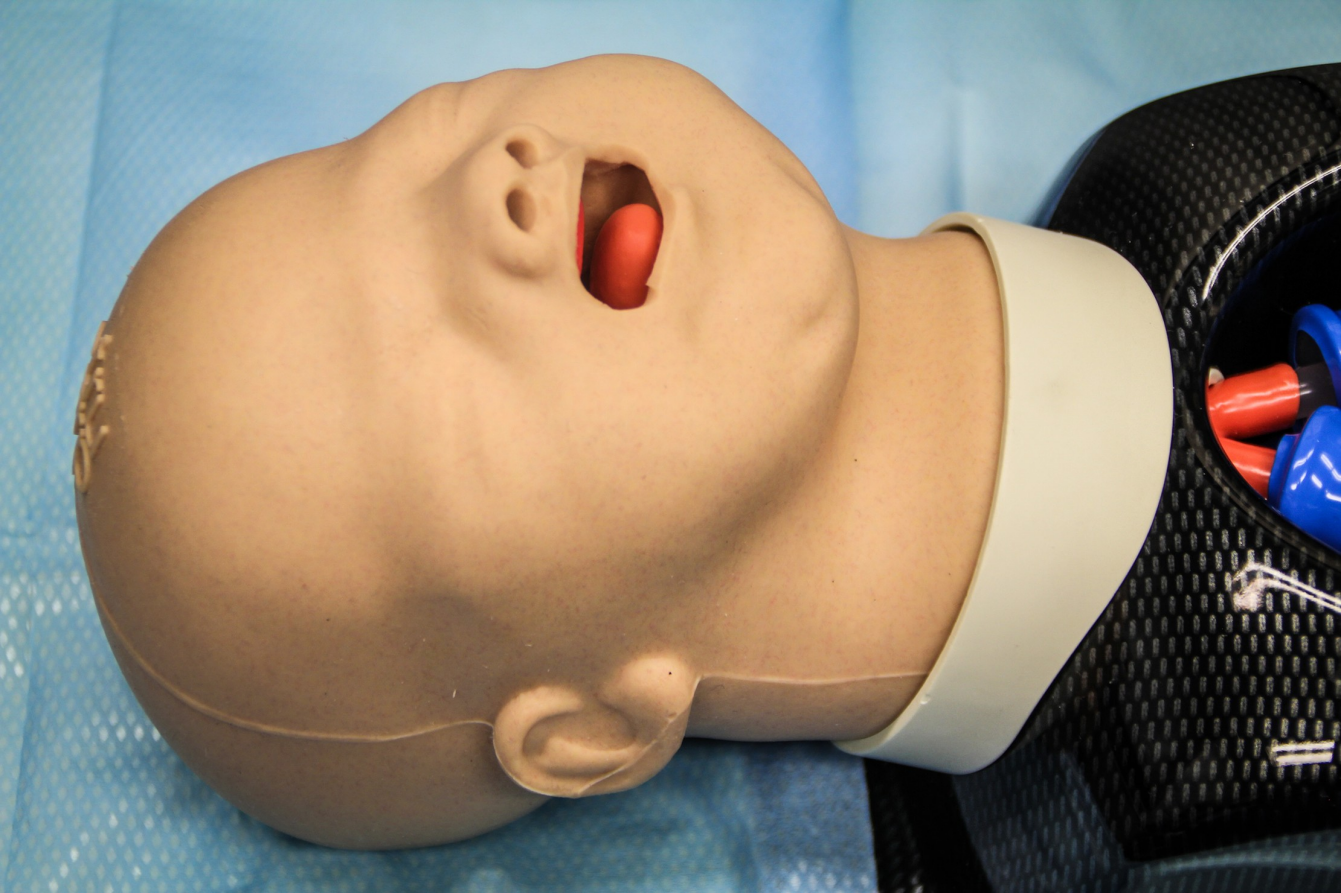
AirSim^®^ Pierre Robin X manikin. The manikin represents the anatomically correct airway of an infant with Pierre Robin syndrome.

**Fig 3:**
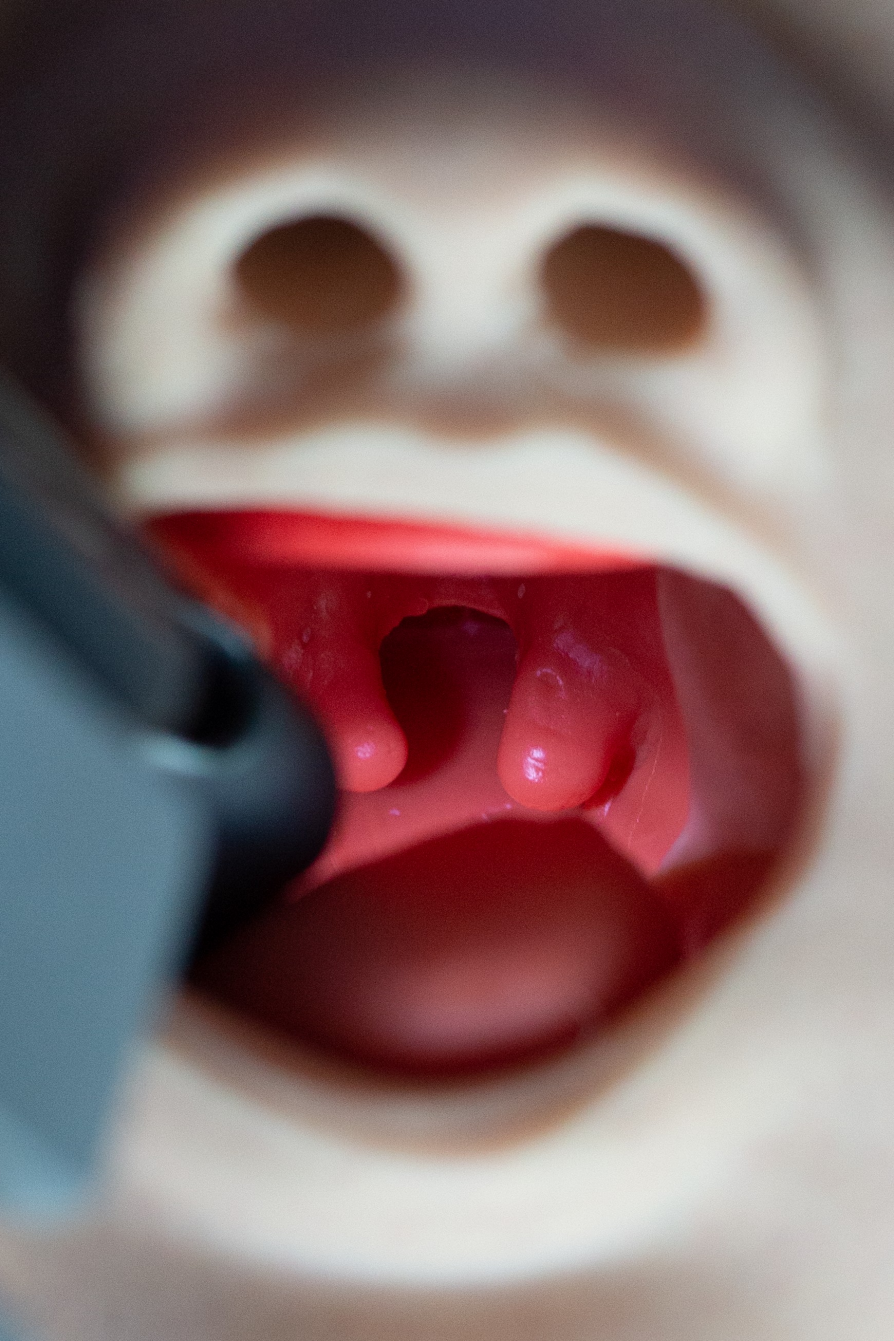
Oropharyngeal view of the AirSim^®^ Pierre Robin X manikin. The anatomically correct airway of the manikin exhibits various congenital defects of an infant with Pierre Robin sequence including micrognathia, glossoptosis, cleft palate and bifid uvula.

Each participant performed endotracheal intubation with the new Glidescope^®^ Core^™^ 10 VL with a hyperangulated LoPro S1 single-use blade (Verathon Medical Canada ULC, Burnaby, BC, Canada), the C-MAC^®^ 8403 ZX VL with a single-use C-MAC S video laryngoscope blade Miller size 0 (Karl Storz, Tuttlingen, Germany) and a conventional Miller laryngoscope blade size 0 (Rüsch^®^ Polaris^™^ Single-Use Laryngoscope Blade Miller 0, Teleflex Medical Europe Ltd, Athlone, Ireland; Heine F.O. SLIM LED metallic laryngoscope handle, Heine Optotechnik GmbH & Co. KG, Gilching, Germany) (Fig 1) in a difficult infant airway manikin (AirSim^®^ Pierre Robin X manikin, TruCorp Ltd, Lurgan, Northern Ireland) (Fig 2). The AirSim^®^ Pierre Robin X manikin has been designed in accordance with real computed tomography data and represents the anatomically correct airway of an infant with PRS (Fig 3).

Randomized order of application was provided by three sealed opaque envelopes each containing a different device name.

All intubations were performed with a 3.5 mm uncuffed endotracheal tube (ETT; pediatric soft endotracheal tube, I.D. 3.5 mm, E.D. 5.2 mm, Vygon, Aachen, Germany). A 2.6 mm outer diameter Rüsch^®^ reusable intubation stylet (Teleflex medical GmbH, Fellbach, Germany) was used to aid intubation with each device.

### Measurements

#### Objective findings

The primary endpoint was the “time to intubate”. Esophageal intubation, attempts requiring more than 120 seconds or more than two attempts (withdrawal of the device from the mouth followed by repositioning) were recorded as failure to intubate. If no tracheal intubation and/or ventilation occurred within 120 s, the “time to intubate” and/or “time to ventilate” were regarded as 120 seconds. In case of an esophageal intubation, the intubation attempt was stopped immediately and no further attempt was allowed. As we could not record either the “time to intubate” or the “time to ventilate”, esophageal intubations were not included in the statistical analysis of the intubation times. Stopwatch studies were made by a single person having direct observation to avoid interobserver error.

In order to compare the different intubation devices, the intubation process was divided into different time episodes:

1. The time to visualization of the glottis (“time to vocal cords”) was defined as the time from insertion of the blade between the teeth until the glottis was visualized.
2. The time to tracheal intubation (“time to intubate”) was defined as the time from insertion of the blade between the teeth until the ETT was deemed to be positioned correctly by each participant.
3. The duration of a successful intubation attempt was defined as the time from insertion of the blade between the teeth until the ETT was connected to a self-inflating resuscitation bag and lung inflation was confirmed (“time to ventilate”).

We recorded the rate of successful intubation, the number of intubation attempts, the number of optimization maneuvers (readjustment of the head position, application of external laryngeal pressure and the need for assistance by a second person), the severity of potential dental trauma, which was visually graded by the pressure exerted on the upper gum line (0 = none, 1 = mild: the blade of the device touched the upper gum line, 2 = moderate: the blade of the device bent the upper gum line, 3 = severe: the blade of the device bent the upper gum line and the upper lip) and the laryngeal view according to the Cormack-Lehane score [15].

#### Subjective findings

After completing the procedure, each anesthetist was asked to score

- the view
- the handling
- the stability
- the force applied during tracheal intubation
- the difficulty of tracheal intubation

using a numeric rating scale (NRS) (0 to 100 mm, from excellent/very easy to poor/very difficult). Finally, the participants were asked to indicate their preferred intubation device.

### Data analysis

The sample size was calculated using G*Power (Version 3.1.9.4, Faul F., 2019, Germany). Based on “time to intubate”-duration compiled in a pilot study, an effect size f of 0.398 was anticipated. Considering an α error of 0.05 and β error of 0.1, sample size calculation indicated that at least 43 participants with limited (senior house officers) and 43 participants with extensive experience (specialist registrars and consultants) would be required.

All the statistical analyses were performed using SPSS software (IBM^®^ SPSS^®^ Statistics, version 26.0, IBM Corp., Armonk, NY, USA). Data for the rate of successful intubation were analyzed using the Cochran`s Q test followed by Dunn-Bonferroni post-hoc tests. Data for the “time to vocal cords”, the “time to intubate”, the “time to ventilate”, the number of intubation attempts, the number of optimization maneuvers, the severity of dental trauma, the Cormack-Lehane score, the view, the handling, the stability, the force applied during tracheal intubation and the difficulty of tracheal intubation were analyzed using paired non-parametric tests. The Friedman and Wilcoxon signed-rank tests with Bonferroni correction were used for multiple and post-hoc comparisons respectively. Statistical significance was accepted at p < 0.05.

## Results

### Participant characteristics

Forty-three anesthetists with limited experience (43 [100%] senior house officers) and forty-three anesthetists with extensive experience (16 [37%] specialist registrars, 27 [63%] consultants) participated in this randomized crossover trial. The participant characteristics, the documented total experience in video laryngoscopy (including assisted and supervised video laryngoscopic intubations), the number of self-performed video laryngoscopic and fiberoptic-guided intubations and the documented previous experience in adult and pediatric anesthesia are summarized in Table 1. The data were retrieved from the electronic patient data management system (NarkoData; IMESO, Hüttenberg, Germany) and cover the last 15 years. Data in the results section are presented as median (inter-quartile range, IQR).

**Table 1:** Participant characteristics and documented previous experience.

	Anesthetists with limited experience	Anesthetists with extensive experience
Gender, n/N (%)		
Female	15/43 (35)	15/43 (35)
Male	28/43 (65)	28/43 (65)
Age (y), median (IQR)	31 (29–34)	42 (40–48)
Clinical experience (y), median (IQR)	3 (1–5)	15 (12–20)
Total experience in video laryngoscopy (n), median (IQR)	36 (23–51)	75 (50–106)
Number of self-performed video laryngoscopic intubations (n), median (IQR)	19 (12–25)	33 (16–60)
Number of self-performed fiberoptic-guided intubation (n), median (IQR)	14 (6–19)	46 (26–63)
Total number of anesthesia procedures (n), median (IQR)	1517 (928–2428)	6722 (4748–9924)
Number of self-performed anesthesia procedures in children under 5 years of age (n), median (IQR)	48 (26–58)	194 (111–370)

Data are presented as median (inter-quartile range, IQR) or as fraction, n/N (%).

### Anesthetists with limited previous experience

Regarding the primary endpoint (“time to intubate”) and the “time to ventilate”, no significant differences were observed between the conventional Miller laryngoscope, the C-MAC^®^ Miller and the Glidescope^®^ Core^™^. However, the Glidescope^®^ Core^™^ enabled a significantly shorter “time to vocal cords” (p < 0.001, 7 [4–11] vs. 12 [7–18], Glidescope^®^ Core^™^ vs. Miller laryngoscope; p < 0.01, 7 [4–11] vs. 10 [7–15], Glidescope^®^ Core^™^ vs. C-MAC^®^ Miller) and significantly improved the Cormack and Lehane grade (p < 0.001, 1 [1–1] vs. 2 [1–2], Glidescope^®^ Core^™^ vs. Miller laryngoscope; p < 0.001, 1 [1–1] vs. 1 [1–2], Glidescope^®^ Core^™^ vs. C-MAC^®^ Miller). In addition, the severity of dental trauma was lower with the Glidescope^®^ Core^™^ (p < 0.001, 0 [0–1] vs. 2 [2–3], Glidescope^®^ Core^™^ vs. Miller laryngoscope; p < 0.001, 0 [0–1] vs. 1 [1–2], Glidescope^®^ Core^™^ vs. C-MAC^®^ Miller). The C-MAC^®^ Miller caused less dental trauma (p < 0.001, 1 [1–2] vs. 2 [2–3], C-MAC^®^ Miller vs. Miller laryngoscope) and improved the Cormack and Lehane grade (p < 0.05, 1 [1–2] vs. 2 [1–2], C-MAC^®^ Miller vs. Miller laryngoscope) compared to the Miller laryngoscope when used by anesthetists with limited previous experience. Although post-hoc analysis revealed no significant differences in the number of intubation attempts and the overall success rate, the failure rate was 11.6% with the Glidescope^®^ Core^™^ and 7% with the Miller laryngoscope. Using the C-MAC^®^ Miller, the overall success rate was 100%. In addition, the number of optimization maneuvers was lower when using the C-MAC^®^ Miller compared to the Glidescope^®^ Core^™^ (p < 0.05, 0 [0–1] vs. 0 [0–1], C-MAC^®^ Miller vs. Glidescope^®^ Core^™^).

Regarding the subjective values, the conventional Miller laryngoscope was considered the most stable of the three devices (p < 0.01, 0.9 [0–1.9] vs. 2 [0.1–3.1], Miller laryngoscope vs. Glidescope^®^ Core^™^; p < 0.05, 0.9 [0–1.9] vs. 2 [0.9–2.9], Miller laryngoscope vs. C-MAC^®^ Miller). However, the Glidescope^®^ Core^™^ and the C-MAC^®^ Miller enabled a significantly better view (p < 0.001, 0.9 [0–1.1] vs. 4 [2–6], Glidescope^®^ Core^™^ vs. Miller laryngoscope; p < 0.001, 2 [1–3] vs. 4 [2–6], C-MAC^®^ Miller vs. Miller laryngoscope) and required less force during tracheal intubation (p < 0.001, 1.8 [1–3] vs. 4 [3–6.9], Glidescope^®^ Core^™^ vs. Miller laryngoscope; p < 0.01, 2.9 [1.1–4.9] vs. 4 [3–6.9], C-MAC^®^ Miller vs. Miller laryngoscope) compared to the conventional Miller laryngoscope. The Glidescope^®^ Core^™^ offered the best view (p < 0.01, 0.9 [0–1.1] vs. 2 [1–3], Glidescope^®^ Core^™^ vs. C-MAC^®^ Miller) and required the least amount of force during tracheal intubation (p < 0.05, 1.8 [1–3] vs. 2.9 [1.1–4.9], Glidescope^®^ Core^™^ vs. C-MAC^®^ Miller). Post-hoc comparison revealed no significant differences for the handling and the difficulty of tracheal intubation.

Forty-nine percent of the anesthetists preferred the C-MAC^®^ Miller. The Glidescope^®^ Core^™^ was preferred by 42% and only 7% of the anesthetists with limited previous experience would use the conventional standard Miller laryngoscope. One participant (2%) could not find any difference.

The objective and subjective findings are summarized in Table 2.

**Table 2:** Intubation data of the anesthetists with limited previous experience.

		Miller blade	C-Mac^®^ Miller	Glidescope^®^ Core^™^
**Overall success rate, n/N (%)**		40/43 (93)	43/43 (100)	38/43 (88.4)
** Esophageal intubation, n (%)**		0 (0)	0 (0)	1 (2.3)
** Prolonged intubation (>120s; > two attempts), n (%)**		3 (7)	0 (0)	4 (9.3)
**Time to vocal cords (s), median (IQR)**		12 (7–18)	10 (7–15)	7 (4–11)^###^ ^§§^
**Time to intubate (s), median (IQR)**		24 (14–32)	22.5 (17–35)	25 (15–57)
**Time to ventilate (s), median (IQR)**		29 (20–41)	30 (23–47)	36.5 (25.4–65.3)
**Number of intubation attempts, n (%)**				
** 1**		41 (95.3)	42 (97.7)	37 (86)
** 2**		1 (2.3)	1 (2.3)	5 (11.6)
** ≥3**		1 (2.3)	0 (0)	1 (2.3)
** Median (IQR)**		1 (1–1)	1 (1–1)	1 (1–1)
**Severity of dental trauma, n (%)**				
** none**		0 (0)	1 (2.3)	29 (67.4)
** mild**		10 (23.3)	22 (51.2)	14 (32.6)
** moderate**		16 (37.2)	17 (39.5)	0 (0)
** severe**		17 (39.5)	3 (7)	0 (0)
** Median (IQR)**		2 (2–3)	1 (1–2) ***	0 (0–1) ^###^ ^§§§^
**Number of optimization maneuvers, n (%)**				
** 0**		26 (60.5)	30 (69.8)	23 (53.5)
** 1**		15 (34.9)	11 (25.6)	13 (30.2)
** ≥2**		2 (4.7)	2 (4.7)	7 (16.3)
** Median (IQR)**		0 (0–1)	0 (0–1) ^§^	0 (0–1)
**View (cm), median (IQR)**		4 (2–6)	2 (1–3) ***	0.9 (0–1.1)^###^ ^§§^
**Handling (cm), median (IQR)**		3 (1.1–5)	2 (1–4)	3 (1–6)
**Stability (cm), median (IQR)**		0.9 (0–1.9) ^##^ *	2 (0.9–2.9)	2 (0.1–3.1)
**Force applied during tracheal intubation (cm), median (IQR)**		4 (3–6.9)	2.9 (1.1–4.9) **	1.8 (1–3) ^###^ ^§^
**Difficulty of tracheal intubation (cm), median (IQR)**		4 (2.1–6.4)	3.5 (2–5)	2.9 (1.9–6.4)
**Cormack-Lehane score, n (%)**				
** 1**		12 (27.9)	25 (58.1)	40 (93)
** 2**		29 (67.4)	18 (41.9)	3 (7)
** 3**		2 (4.7)	0 (0)	0 (0)
** 4**		0 (0)	0 (0)	0 (0)
** Median (IQR)**		2 (1–2)	1 (1–2) *	1 (1–1) ^###^ ^§§§^
**Preferred laryngoscope, n/N (%)**	no difference 1/43 (2.3)	3/43 (7)	21/43 (48.8)	18/43 (41.9)

Data are presented as median (inter-quartile range, IQR), number n (%) or as fraction n/N (%). Subjective findings are presented as numeric rating scale values (0 to 10 cm, from excellent/very easy to poor/very difficult).

* p < 0.05 C-MAC^®^ vs. Miller blade

** p < 0.01 C-MAC^®^ vs. Miller blade

*** p < 0.001 C-MAC^®^ vs. Miller blade

^###^ p < 0.001 Glidescope^®^ Core^™^ vs. Miller blade

^§^ p < 0.05 Glidescope^®^ Core^™^ vs. C-MAC^®^

^§§^ p < 0.01 Glidescope^®^ Core^™^ vs. C-MAC^®^

^§§§^ p < 0.001 Glidescope^®^ Core^™^ vs. C-MAC^®^.

### Anesthetists with extensive previous experience

In the hands of anesthetists with extensive previous experience the Glidescope^®^ Core^™^ enabled a significantly shorter “time to vocal cords” (p < 0.001, 5.8 [4.7–7.8] vs. 8.2 [6–12], Glidescope^®^ Core^™^ vs. Miller laryngoscope; p < 0.05, 5.8 [4.7–7.8] vs. 7 [5.6–10], Glidescope^®^ Core^™^ vs. C-MAC^®^ Miller). However, the “time to intubate” and the “time to ventilate” were significantly increased using the Glidescope^®^ Core^™^ compared to the conventional Miller laryngoscope and the C-MAC^®^ Miller (“time to intubate”: p < 0.001, 36 [17–71] vs. 19 [14.9–27.1], Glidescope^®^ Core^™^ vs. Miller laryngoscope; p < 0.001, 36 [17–71] vs. 15.9 [11.1–22], Glidescope^®^ Core^™^ vs. C-MAC^®^ Miller; “time to ventilate”: p < 0.001, 43 [25–81.3] vs. 24 [19.8–33.3], Glidescope^®^ Core^™^ vs. Miller laryngoscope; p < 0.001, 43 [25–81.3] vs. 21 [17–29], Glidescope^®^ Core^™^ vs. C-MAC^®^ Miller). In addition, the number of optimization maneuvers was lower when using the Miller laryngoscope and the C-MAC^®^ Miller (p < 0.05, 0 [0–1] vs. 1 [0–1], Miller laryngoscope vs. Glidescope^®^ Core^™^; p < 0.01, 0 [0–0] vs. 1 [0–1], C-MAC^®^ Miller vs. Glidescope^®^ Core^™^). Although post-hoc analysis revealed no significant differences in the number of intubation attempts and the overall success rate, the failure rate was 14% with the Glidescope^®^ Core^™^, 4.7% with the conventional Miller laryngoscope and only 2.3% with the C-MAC^®^ Miller. The use of video laryngoscopy significantly reduced the severity of dental trauma (p < 0.001, 0 [0–0] vs. 2 [2–2], Glidescope^®^ Core^™^ vs. Miller laryngoscope; p < 0.001, 1 [1–1] vs. 2 [2–2], C-MAC^®^ Miller vs. Miller laryngoscope). However, the Glidescope^®^ Core^™^ caused even less dental trauma than the C-MAC^®^ Miller (p < 0.001, 0 [0–0] vs. 1 [1–1], Glidescope^®^ Core^™^ vs. C-MAC^®^ Miller). Both video laryngoscopes significantly improved the Cormack and Lehane grade compared to the conventional Miller laryngoscope (p < 0.001, 1 [1-1] vs. 2 [1–2], Glidescope^®^ Core^™^ vs. Miller laryngoscope; p < 0.01, 1 [1-2] vs. 2 [1–2], C-MAC^®^ Miller vs. Miller laryngoscope).

Regarding the subjective values, the Glidescope^®^ Core^™^ and the C-MAC^®^ Miller enabled a significantly better view (p < 0.001, 0.6 [0-1] vs. 3 [1.8–5], Glidescope^®^ Core^™^ vs. Miller laryngoscope; p < 0.001, 1 [0-2] vs. 3 [1.8–5], C-MAC^®^ Miller vs. Miller laryngoscope) and required less force during tracheal intubation (p < 0.001, 1.1 [0.5-2.2] vs. 3.1 [2–5], Glidescope^®^ Core^™^ vs. Miller laryngoscope; p < 0.001, 1 [0.4-3] vs. 3.1 [2–5], C-MAC^®^ Miller vs. Miller laryngoscope). In addition, the C-MAC^®^ Miller significantly facilitated tracheal intubation compared to the conventional Miller laryngoscope (p < 0.001, 2 [1-3] vs. 3 [2–5], C-MAC^®^ Miller vs. Miller laryngoscope). Post-hoc comparison revealed no significant differences for the handling and the stability.

Fifty-one percent of the anesthetists preferred the C-MAC^®^ Miller. The Glidescope^®^ Core^™^ was preferred by 37% and only 12% of the anesthetists with extensive previous experience would use the conventional standard Miller laryngoscope.

The objective and subjective findings are summarized in Table 3.

**Table 3:** Intubation data of the anesthetists with extensive previous experience.

		Miller blade	C-Mac^®^ Miller	Glidescope^®^ Core^™^
**Overall success rate, n/N (%)**		41/43 (95.3)	42/43 (97.7)	37/43 (86)
** Esophageal intubation, n (%)**		1 (2.3)	0 (0)	0 (0)
** Prolonged intubation (>120s; > two attempts), n (%)**		1 (2.3)	1 (2.3)	6 (14)
**Time to vocal cords (s), median (IQR)**		8.2 (6–12)	7 (5.6–10)	5.8 (4.7–7.8) ^###^^§^
**Time to intubate (s), median (IQR)**		19 (14.9–27.1)^###^	15.9 (11.1–22)^§§§^	36 (17–71)
**Time to ventilate (s), median (IQR)**		24 (19.8–33.3)^###^	21 (17–29) ^§§§^	43 (25–81.3)
**Number of intubation attempts, n (%)**				
** 1**		40 (93)	41 (95.3)	36 (83.7)
** 2**		3 (7)	1 (2.3)	7 (16.3)
** ≥3**		0 (0)	1 (2.3)	0 (0)
** Median (IQR)**		1 (1–1)	1 (1–1)	1 (1–1)
**Severity of dental trauma, n (%)**				
** none**		1 (2.3)	1 (2.3)	34 (79.1)
** mild**		2 (4.7)	33 (76.7)	9 (20.9)
** moderate**		30 (69.8)	7 (16.3)	0 (0)
** severe**		10 (23.3)	2 (4.7)	0 (0)
** Median (IQR)**		2 (2–2)	1 (1–1) ******	0 (0–0) ^###^^§§§^
**Number of optimization maneuvers, n (%)**				
** 0**		29 (67.4)	35 (81.4)	19 (44.2)
** 1**		13 (30.2)	7 (16.3)	18 (41.9)
** ≥2**		1 (2.3)	1 (2.3)	6 (14)
** Median (IQR)**		0 (0–1) ^#^	0 (0–0) ^§§^	1 (0–1)
**View (cm), median (IQR)**		3 (1.8–5)	1 (0–2) ***	0.6 (0–1)^###^
**Handling (cm), median (IQR)**		2 (0.8–4)	1 (0–2.5)	2 (0.9–5)
**Stability (cm), median (IQR)**		0.6 (0–1.2)	0.9 (0–1.9)	1 (0.2–2)
**Force applied during tracheal intubation (cm), median (IQR)**		3.1 (2–5)	1 (0.4–3) ***	1.1 (0.5–2.2) ^###^
**Difficulty of tracheal intubation (cm), median (IQR)**		3 (2–5)	2 (1–3) ***	3.5 (1–5)
**Cormack-Lehane score, n (%)**				
** 1**		19 (44.2)	32 (74.4)	38 (88.4)
** 2**		22 (51.2)	11 (25.6)	4 (9.3)
** 3**		2 (4.7)	0 (0)	1 (2.3)
** 4**		0 (0)	0 (0)	0 (0)
** Median (IQR)**		2 (1–2)	1 (1–2) **	1 (1–1) ^###^
**Preferred laryngoscope, n/N (%)**		5/43 (11.6)	22/43 (51.2)	16/43 (37.2)

Data are presented as median (inter-quartile range, IQR), number n (%) or as fraction n/N (%). Subjective findings are presented as numeric rating scale values (0 to 10 cm, from excellent/very easy to poor/very difficult).

** p < 0.01 C-MAC^®^ vs. Miller blade

*** p < 0.001 C-MAC^®^ vs. Miller blade

^#^ p < 0.05 Glidescope^®^ Core^™^ vs. Miller blade

^###^ p < 0.001 Glidescope^®^ Core^™^ vs. Miller blade

^§^ p < 0.05 Glidescope^®^ Core^™^ vs. C-MAC^®^

^§§^ p < 0.01 Glidescope^®^ Core^™^ vs. C-MAC^®^

^§§§^ p < 0.001 Glidescope^®^ Core^™^ vs. C-MAC^®^.

## Discussion

In the last decade, video laryngoscopy has become an important and commonly used tool for the management of difficult airway in pediatric patients, including infants and neonates [16]. In addition, video laryngoscopy has also been shown to be an effective tool in syndromic children with potential difficult airways [10, 11, 17, 18]. However, evidence to guide the choice of the most appropriate VL for airway management in children with craniofacial anomalies, such as PRS, is insufficient. Thus, the aim of our prospective randomized crossover study was to compare the performance of the new Glidescope^®^ Core^™^ VL with its hyperangulated LoPro S1 blade, the nonangulated C-MAC^®^ Miller VL and the conventional Miller laryngoscope when used by anesthetists with limited and extensive previous experience in a simulated Pierre Robin sequence.

The results of our study suggest that in the hands of anesthetists with limited and extensive experience, the Glidescope^®^ Core^™^ and the C-MAC^®^ Miller provide superior intubation conditions, including a significantly better view of the laryngeal inlet, less force during tracheal intubation and, consequently, less severity of dental trauma. The hyperangulated Glidescope^®^ Core^™^ enabled the best glottic view, caused the least dental trauma and significantly decreased the “time to vocal cords”, in both groups. This might be due to the greater angulation of the LoPro S1 blade, which offers a ‘view around the corner’ and enables optimal glottis visualization via the camera, without the need to align the oral, pharyngeal and tracheal axes [19]. In a randomized crossover manikin-based study, Godai and colleagues could demonstrate that the MultiViewScope Stylet Scope, a video laryngoscope system including a handle with integrated monitor and a rigid, angulated stylet scope, improved the force exerted on the incisors during tracheal intubation, the Cormack and Lehane glottic view and the difficulty of tracheal intubation in a simulated difficult pediatric airway when used by expert anesthesiologists and anesthesiology residents [20]. The nonangulated C-MAC^®^ Miller also improved glottic exposure in our simulated Pierre Robin sequence compared to the conventional Miller laryngoscope. This is in line with the findings of Hackell and colleagues. In a case series of seven infants with difficult airways, the Cormack and Lehane glottic view was improved to 1-2 using a nonangulated VL [21]. In addition, in a prospective, randomized study, including 56 children younger than four years of age with normal airways, video laryngoscopy with a nonangulated Miller blade provided an improved view of the glottis compared to conventional direct laryngoscopy [22].

Although the superior viewing angle of the hyperangulated LoPro blade of the Glidescope^®^ Core^™^ improved the Cormack and Lehane glottic view, the acute distal blade angulation together with the inherent anatomical complexity of the simulated PRS caused difficulties in converting the adequate view to successful endotracheal intubation. Without having to align the oral, pharyngeal and tracheal axes for optimal visualization, it might be difficult to direct the endotracheal tube toward the glottis and through the plane of the vocal cords. Thus, the failure rate for tracheal intubation with the Glidescope^®^ Core^™^ was 14% and 11.6% when used by anesthetists with extensive and limited previous experience in the simulated Pierre Robin sequence. This is in line with the findings of Park and colleagues who analyzed data from the pediatric difficult intubation registry and found that in children weighing less than 10 kg, the likelihood of successful intubation when using the Glidescope^®^ in the setting of a Cormack-Lehane grade 1 or 2a views was only 53% [23]. In addition, in a retrospective observational study, analyzing data submitted by 28 institutions into the pediatric difficult intubation registry from March 2017 to January 2020, Peyton and colleagues could demonstrate that in infants weighing less than 5 kg, video laryngoscopy with standard Macintosh and Miller blades was associated with a significantly greater success rate than video laryngoscopy with non-standard blades [24]. Zhang and colleagues also showed that in pediatric patients less than 6 years of age more than half of all endotracheal intubations with the Glidescope^®^ VL were associated with technical difficulties. Their observed first-attempt intubation success rate was only 80% [25].

Regarding the overall success rate, the C-MAC^®^ Miller performed best. The overall success rate with the nonangulated C-MAC^®^ Miller was 100% when used by anesthetists with limited previous experience. Compared to the Glidescope^®^ Core^™^ and the conventional Miller laryngoscope, this corresponds to an absolute risk reduction of 11.6% and 7%, respectively. In the hands of anesthetists with extensive previous experience, C-MAC^®^ Miller also decreased the failure rate for tracheal intubation from 14% with the Glidescope^®^ Core^™^ and 4.7% with the conventional Miller laryngoscope to 2.3%. Although hyperangulated VLs have been shown to be beneficial for difficult airway management in adult patients [26], Saracoglu and colleagues found that the nonangulated C-MAC^®^ Miller was advantageous over the hyperangulated McGrath VL in simulated difficult pediatric airway [27]. The results of our study suggest that in Pierre Robin sequence the nonangulated C-MAC^®^ Miller could facilitate the correct placement of the endotracheal tube and may increase the overall success rate of intubation compared to the hyperangulated Glidescope^®^ Core^™^. This might be due to the craniofacial characteristics and the unique difficult airway of infants with PRS, including micrognathia and glossoptosis with limited pharyngeal space. These characteristics together with the anteriorly angled larynx of infants can cause difficulties in inserting the endotracheal tube with a hyperangulated VL despite an optimal glottic view.

In the hands of anesthetists with limited experience, we could not determine any significant differences for either the "time to intubate" or the "time to ventilate". However, for anesthetists with extensive experience, the “time to intubate” and the “time to ventilate” were significantly increased using the Glidescope^®^ Core^™^. The specialist registrars and the consultants included in our study are most familiar with and used to direct pediatric laryngoscopy in daily practice. In addition, the ratio of direct laryngoscopy with the Miller blade to video laryngoscopy is significantly shifted toward direct laryngoscopy despite the higher total number of video laryngoscopies compared with the less experienced anesthetists. Therefore, switching to an indirect intubation technique with a hyperangulated VL could lead to major technical difficulties in endotracheal tube placement despite superior glottic exposure. This might explain the higher number of optimization maneuvers and the increased failure rate when using the Glidescope^®^ Core^™^. In addition, anesthetists experienced in direct laryngoscopy can identify landmarks of the anatomy and may be able to perform fast and successful tracheal intubation with the conventional Miller laryngoscope even in case of worse Cormack and Lehane grade. Nonetheless, a greater peak force with increased severity of dental trauma was required to align the axes and to visualize the glottis. The nonangulated C-MAC^®^ Miller combines the advantages of both usual direct and video laryngoscopy and thus facilitated tracheal intubation compared to the conventional Miller laryngoscope when used by anesthetists with extensive previous experience.

In our prospective randomized crossover study, the C-MAC^®^ Miller performed best. The overall preference of the participants confirmed these results. Fifty-one percent of the anesthetists with extensive previous experience and forty-nine percent of the anesthetists with limited previous experience preferred the nonangulated C-MAC^®^ Miller.

### Limitations

The results of this study need to be interpreted with consideration of certain limitations. First, the study was conducted using an infant high-fidelity manikin and not on patients. Although manikins do not fully resemble human structures, the use of anatomically correct manikins has proven to be a reliable surrogate for the clinical context [28]. The airway of the AirSim^®^ Pierre Robin X manikin has been designed in accordance with real computed tomography data and has been evaluated in clinical studies repeatedly [14, 20, 29]. In addition, clinical investigations such as the comparison of different intubation devices in infant PRS patients are hardly feasible due to the low incidence and ethical aspects. Second, the potential for bias exists, as it was not possible to blind the participants or the assessors to the airway device used. Third, the anesthetists were aware that their performance was being assessed, which could lead to an altered performance due to the Hawthorne effect [30]. Fourth, certain measurements used in this study, such as grading the force applied during tracheal intubation, have a subjective nature. Fifth, although the Glidescope^®^ Core^™^ was associated with a statistically significant reduction in the “time to vocal cords” when used by anesthetists with limited and anesthetists with extensive previous experience, the clinical impact of this time difference remains uncertain. Sixth, a malleable intubation stylet was used to facilitate endotracheal intubation. Although a malleable stylet enables the shape of the endotracheal tube to be adapted to the respective anatomical conditions, the flexibility of the stylet could affect the performance to some extent, and therefore the results might have been different if a pre-shaped rigid stylet had been used for indirect laryngoscopy. However, in a randomized clinical trial, Turkstra and colleagues demonstrated that a rigid styled offered no significant advantage over the standard malleable stylet for orotracheal intubation with the Glidescope^®^ when used by experienced operators [31]. In addition, Jones and colleagues showed that the GlideRite^®^ rigid stylet and the standard malleable stylet have similar performance characteristics when used by inexperienced operators for GlideScope^®^-assisted orotracheal intubation [32]. Seventh, the gender disparity in the present study might have influenced our results. However, Waddington and colleagues could demonstrate that female and male intubators did not differ in their ability to intubate or in the forces they exerted during tracheal intubation of an airway management trainer [33]. Finally, we compared only two different VLs and one conventional Miller laryngoscope. There are other types of VLs and their utility in the airway management of pediatric patients with PRS might be different and should be investigated. In addition, conventional Miller blades for direct laryngoscopy can vary substantially and therefore external validity may be limited to some extent.

## Conclusions

Both hyperangulated and nonangulated VLs provided superior intubation conditions including a better visualization of the glottis, less force during tracheal intubation and less severity of dental trauma. Although the hyperangulated Glidescope^®^ Core^™^ enabled the best glottic exposure, caused the least dental trauma and significantly decreased the “time to vocal cords”, the Glidescope^®^ Core^™^ was associated with the highest failure rate of intubation in both groups and an increased “time to intubate” and “time to ventilate” when used by anesthetists with extensive previous experience. The nonangulated C-MAC^®^ Miller did not affect the intubation times compared to conventional direct laryngoscopy with the Miller blade. However, the C-MAC^®^ Miller facilitated the correct placement of the endotracheal tube and showed the highest overall success rate in both groups.

Our results therefore suggest that the C-MAC^®^ Miller could be beneficial and may contribute to increased safety in the airway management of infant patients with PRS when used by both anesthetists with limited and extensive previous experience.

## Supporting information

S1 Dataset (SAV)
